# The inverted U‐shaped relationship between epinephrine and pancreatic ductal adenocarcinoma patients' survival with compensation of lymphocyte

**DOI:** 10.1002/cam4.7164

**Published:** 2024-04-04

**Authors:** Bing‐Xue Li, Xiao‐Cen Zhu, Xia‐Xing Deng

**Affiliations:** ^1^ Department of General Surgery, Pancreatic Disease Center, Ruijin Hospital Shanghai Jiaotong University School of Medicine Shanghai China; ^2^ Institute of Pancreatic Diseases Shanghai Jiaotong University School of Medicine Shanghai China; ^3^ State Key Laboratory of Oncogenes and Related Genes Shanghai China; ^4^ Institute of Translational Medicine Shanghai Jiaotong University Shanghai China; ^5^ Shanghai Key Laboratory of Pancreatic Neoplasms Translational Medicine Shanghai China; ^6^ Core Facility of Basic Medical Sciences Shanghai Jiaotong University School of Medicine Shanghai China

**Keywords:** immunology, pancreatic cancer, prognostic factor, survival

## Abstract

**Background:**

The relationship between epinephrine and cancer can be dose‐dependent in in vivo study. Whether it is the same in human body still needs verification.

**Method:**

We used frozen human pancreatic ductal adenocarcinoma (PDAC) tissues to detect epinephrine content and analyzed its relationship with survival using the K‐M method and Cox regression. Disturbance of blood cell count and C‐reactive protein and identification of related potent intermediary factors were also analyzed.

**Results:**

K‐M plot and Cox regression all showed the inverted U‐shaped relationship between epinephrine and PDAC survival. Lymphocyte adjustment can increase the HRs of epinephrine for PDAC death by >10%.

**Conclusion:**

Epinephrine played an anti‐tumor or pro‐tumor effect depending on the specific concentration. Circulating lymphocyte count was elevated and might acted as a compensation pathway to reduce the pro‐tumor effect of epinephrine to PDAC.

## Graphical abstract

Epinephrine played an anti‐tumor or pro‐tumor effect depending on the specific concentration. Circulating lymphocyte count was elevated and might acted as a compensation pathway to reduce the pro‐tumor effect of epinephrine to pancreatic ductal adenocarcinoma.

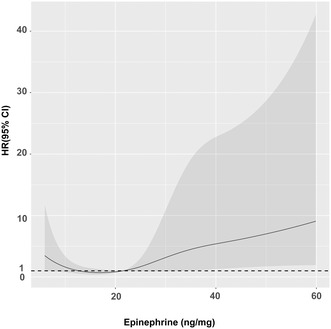

## 1 INTRODUCTION

Pancreatic ductal adenocarcinoma (PDAC) is a highly fatal disease with a median survival time of only 2 years.^1^ Most patients are found to have advanced disease when first diagnosed as PDAC because limited indications are shown in the early stage. Apart from the difficulty of early detection, treatment methods are also limited to surgery and chemotherapy, even though immunotherapy and targeted therapy are developing fast nowadays. It is urgent to find new perspectives to dig into the progression mechanism of PDAC to give guidance to the clinical management of PDAC patients.

Recently the relationship between the autonomic nervous system and PDAC has attracted the attention of researchers. ADRB2‐mediated adrenergic signaling^2^ or and muscarinic receptors‐mediated cholinergic signaling^3^ or nicotinic receptors‐mediated cholinergic signaling^4^ were shown to promote PDAC progression. However, they all only focused on the specific receptors downstream of autonomic signaling. What's more, as the efferent transmitters (epinephrine, norepinephrine, and acetylcholine) hold high polarity, the exact content is hard to get through high throughput metabolomics study. So the overall relationship of autonomic signaling and PDAC has been poorly investigated.

Epinephrine, the only efferent transmitter that holds endocrine action which is different from norepinephrine and acetylcholine, is always regarded as a stress hormone. But the effect of epinephrine on cancer progression may be complex depending on the specific level of it. It seems that low dose of epinephrine can be body protective and inhibit cancer progression while high dose of epinephrine shows the other way.^5^ But this was only proved in the mice model and has not been verified in human related studies.

Circulating blood cells, which are frequently detected in clinical practice, are also related to cancer progression. White blood cells can be divided into two groups: myeloid cells and lymphocytes. They all play complex roles in cancer progression.^6^, ^7^

In this study, we try to analyze the relationship between epinephrine and PDAC progression and explore whether clinically tested blood cells play a part in it.

## 2 METHOD

### 2.1 Patients

We retrospectively assessed 32 patients with historically diagnosed PDAC in Ruijin Hospital and written informed consent was obtained. We included patients that met the following two criteria: (1) historically diagnosed with pancreatic ductal adenocarcinoma from 2021.9.30 to 2021.11.30; (2) with sample collected and kept in Standardized Clinical Biobank in our hospital. All patients with neuroendocrine, duodenal, distal‐bile duct, and ampullary carcinoma were excluded. We also excluded patients who died from postoperative complications. This study was approved by the Institutional Review Board of Ruijin Hospital, Shanghai Jiaotong University School of Medicine.

### 2.2 Clinical data collection

The preoperative laboratory data of blood routine examination and T lymphocyte subsets within 1 week before surgery were all collected. Tumor stage was determined according to American Joint Committee on Cancer TNM staging manual (8th edition). Pathology, perineural invasion, vascular invasion, and margin status were collected from pathology records. Treatment detail (type of surgery, neoadjuvant therapy, and adjuvant therapy), survival status together with time of death or time of last follow‐up were extracted from medical records.

### 2.3 Epinephrine detection

Frozen tissue of human tumor was weighed and then added 10 times the volume of the frozen PBS buffer and 2 mm steel ball. Homogenize the samples in a cryogenic grinder at −20°C with 65 HZ for 5 min. Aliquot 50 μL of homogenized solution into a clean 1.5 mL tube then add 200 μL of frozen acetonitrile, mix the tube with 800 g at 4°C for 10 min then centrifuge the tube for 10 min (4°C, 9600 g). Take 220 μL of the supernatant into another clean 1.5 mL tube, and dry the supernatant on nitrogen. Add 50 μL of cold reconstituted solution (water: methanol,V:V,4:1) then mix well and collect for LC–MS/MS analysis. 2.0 μL of solution was measured using chromatographed by an Agilent 1290 UHPLC system equipped with a 2.1 × 50 mm, 1.8 μM ACQUITY UPLC HSS T3 column (Waters, Milford, MA) at a flow rate of 0.4 mL/min. Solvent A (10 mM ammonium acetate and 0.1% formic acid in water) was held at 98% for 0.8 min, then a linear gradient to 25% solvent B (10 mM ammonium acetate and 0.1% formic acid in acetonitrile) was applied over the next 4.6 min. The column was held at 20% B for 1.0 min and then equilibrated to 98% A for 3.6 min. The needle was washed prior to each injection with a mixed solvent (2% acetonitrile in water). And selective reaction monitoring was performed in positive ion mode using an AB SCIEX 6500 plus QTRAP with the following transition: m/z 166.0/107.0 for epinephrine.

### 2.4 Statistical analysis

Statistical analysis in this study was performed by R 4.2.3 and GraphPad Prism 8.0. Overall survival was calculated from the date of surgery to the date of death. Patients alive at the last follow‐up date were censored in survival analyses. Patients were divided into two groups using the maximally selected rank statistics to get the optimal cut‐off values; and they were divided into three groups with equal numbers (or nearly equal numbers, of which the medium group was 10 patients while the other two groups were 11 patients). Survival rates were generated using the Kaplan–Meier method^8^ and cumulative death risk curves were produced and displayed. Survival curves were compared by log‐rank test. Comparison of differences between two groups were conducted using t test for normally distributed measurement data with homogeneous variance and chi‐square test for dichotomous data or unordered multi‐group categorical data. For ordered categorical data and measurement data that were not normally distributed or the variance of which was not homogeneous, Wilcoxon rank sum test would be performed. Univariate and multivariate Cox regression analyses were performed using the Cox proportional hazards model to scientifically explore the relationship of epinephrine with survival. We also used restricted cubic splines with four knots at the 5th, 35th, 65th, and 95th centiles to flexibly model the association of epinephrine as a continuous variable with mortality and visualize it. *p* < 0.1 was defined as statistically significant.

## 3 RESULTS

### 3.1 Patient characteristics

We selected 32 PDAC patients who underwent surgery whose tissues were frozen and collected in the Standardized Clinical Biobank in Ruijin Hospital. The specific clinical characteristics are as follows (Table 1). Subgroup characteristics were in Table S1 of the supplementary file.

**TABLE 1:** Patient characteristics.

	PDAC patients (*n* = 32)
Age, years	62.69 ± 8.59
Gender	
Male	17 (53.1)
Female	15 (46.9)
BMI, kg/m^2^	22.93 ± 2.83
Pathology	
Poor	6 (18.75)
Well/moderate	26 (81.25)
TNM	
I	4 (12.5)
II	15 (46.875)
III	9 (28.125)
IV	4 (12.5)
Perineural invasion	
Yes	32 (100.0)
No	0 (0)
Vascular invasion	
Yes	22 (68.8)
No	10 (31.2)
Type of surgery	
PD	13 (40.6)
DP	15 (50)
TP	3 (9.4)
MP	1 (3.1)
R	
R0	31 (96.9)
R1	1 (3.1)
CA19‐9, U/mL	214.35 [47.88, 738.55]
While blood cell, 10^9^/L	5.87 [5.10, 7.32]
Neutrophil, 10^9^/L	3.81 [3.17, 5.86]
Platelet, 10^9^/L	193.66 (65.94)
Lymph, 10^9^/L	1.35 [1.14, 1.80]
CD3^+^, %	74.01 (9.01)
CD3^+^CD4^+^, %	45.85 (11.04)
CD3^+^CD8^+^, %	24.07 (6.96)
CD3^+^CD4^+^/CD3^+^CD8^+^	1.90 [1.44, 2.56]
Mono, 10^12^/L	0.40 [0.33, 0.45]
CRP (mg/L)	2.00 [1.15, 7.22]

Abbreviations: BMI, body mass index; CRP, C‐reactive protein; DP, distal pancreatectomy; MP, middle segment pancreatectomy; PD, pancreatoduodenectomy; PDAC, pancreatic ductal adenocarcinoma; TP, total pancreatectomy.

### 3.2 PDAC patients with medium epinephrine levels survived the longest

**FIGURE 1:**
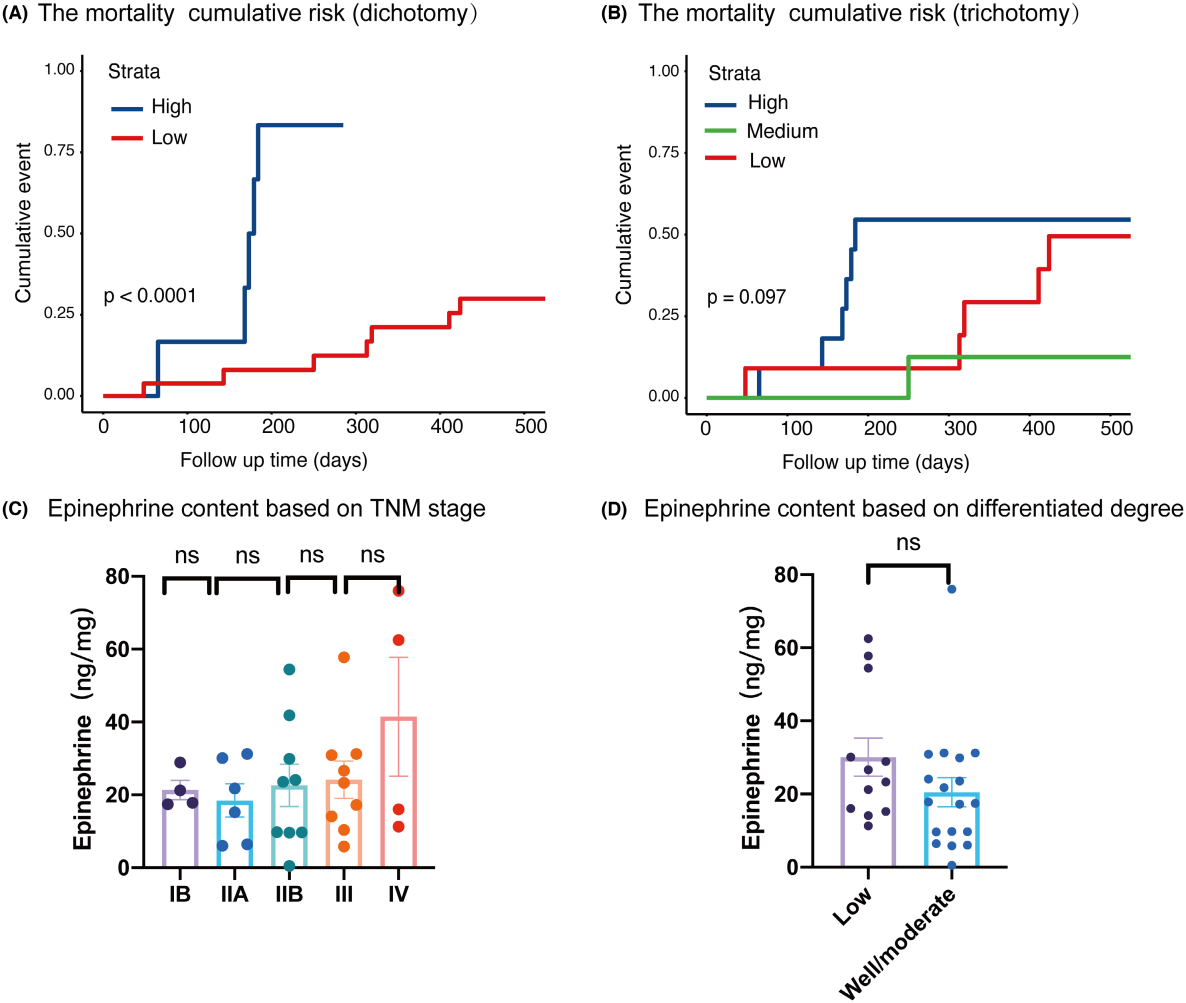
Relationship of epinephrine with mortality cumulative risk. (A) The mortality cumulative risk of epinephrine when divided into 2 groups (>31.21 ng/mg or ≤31.21 ng/mg). (B) The mortality cumulative risk of epinephrine when divided into 3 groups (>27 ng/mg, 16–27 ng/mg or <27 ng/mg). (C) Epinephrine level comparison among different TNM stages. (D) Epinephrine level comparison between low and well/moderate differentiated tumors.

**FIGURE 2:**
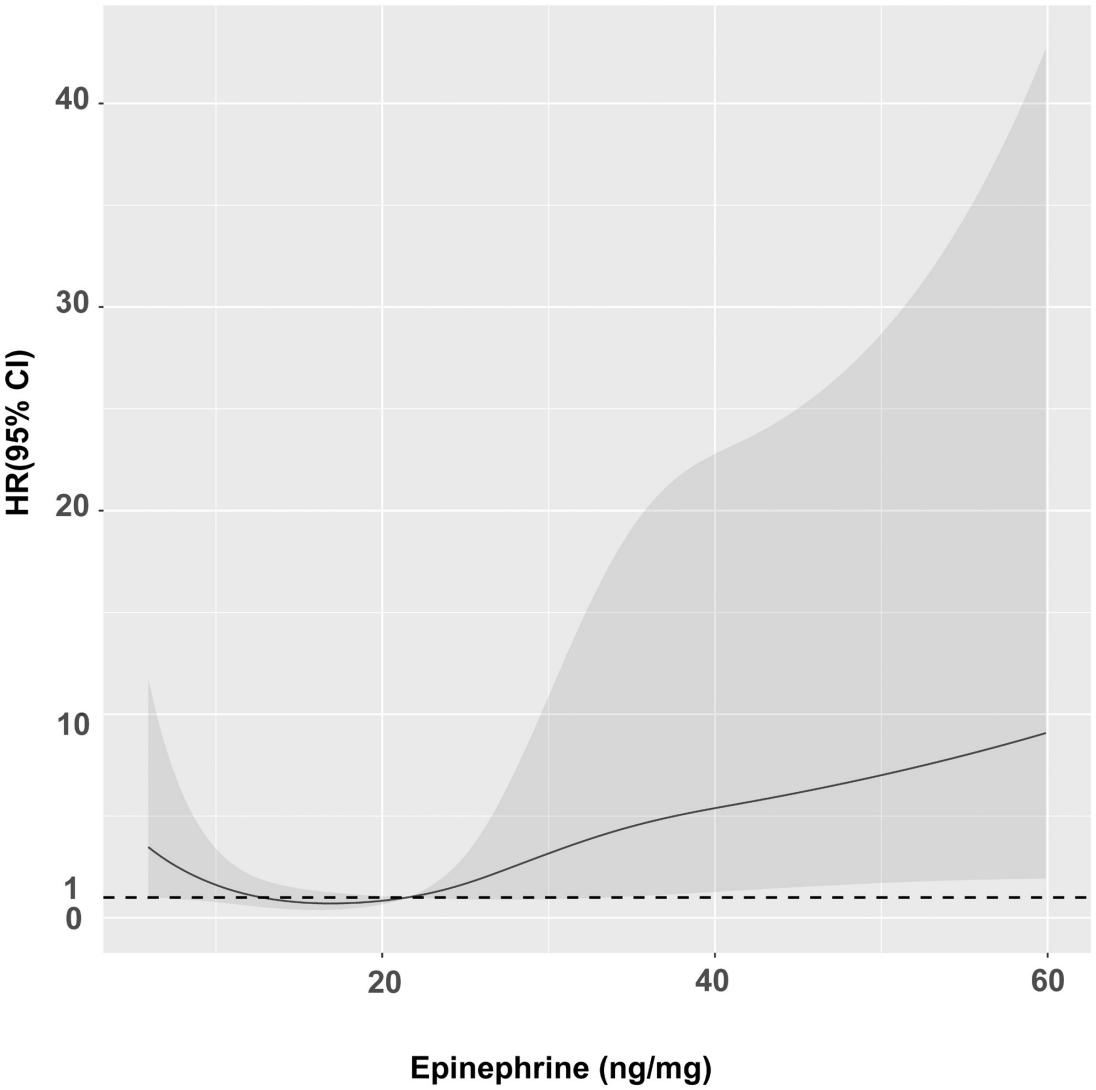
HRs of epinephrine (ng/mg) for PDAC mortality. Analysis was done using restricted cubic splines with four knots at the 5th, 35th, 65th, and 95th centiles. PDAC, pancreatic ductal adenocarcinoma.

In order to analysis the relationship between epinephrine and survival of PDAC patients, cumulative death risk curves were plotted using the K‐M method. As shown in Figure 1A, when we divided epinephrine into 2 groups, the high group (epinephrine >31.21 ng/mg) showed a higher death risk compared with the low group (*p* < 0.0001). And when we divided the patients into three groups (high: >27 ng/mg; medium:16–27 ng/mg; low:<16 ng/mg), the medium group showed the lowest death rate (Figure 2B).

We also compared the epinephrine level between different TNM stages or pathology degrees, and found no correlation between TNM or pathology degree with epinephrine (Figure 1C,D). This indicates that epinephrine correlates with higher death rate independent of cancer stage or pathology degree.

As a previous study has demonstrated that the correlation between epinephrine and survival was not linear, we also used restricted cubic splines with four knots at the 5th, 35th, 65th, and 95th centiles to flexibly model the association of epinephrine with mortality and visualize it. It demonstrated the U‐shaped relationship between epinephrine and mortality (i.e. inverted U‐shaped relationship between epinephrine and survival, *p* for non‐linearity = 0.0331). Epinephrine appeared to be a risk factor for mortality when it was <12.7 ng/mg or >21.7 ng/mg but acted otherwise between 12.7 and 21.7 ng/mg.

### 3.3 High epinephrine accompanies with high circulating lymphocyte count

**FIGURE 3:**
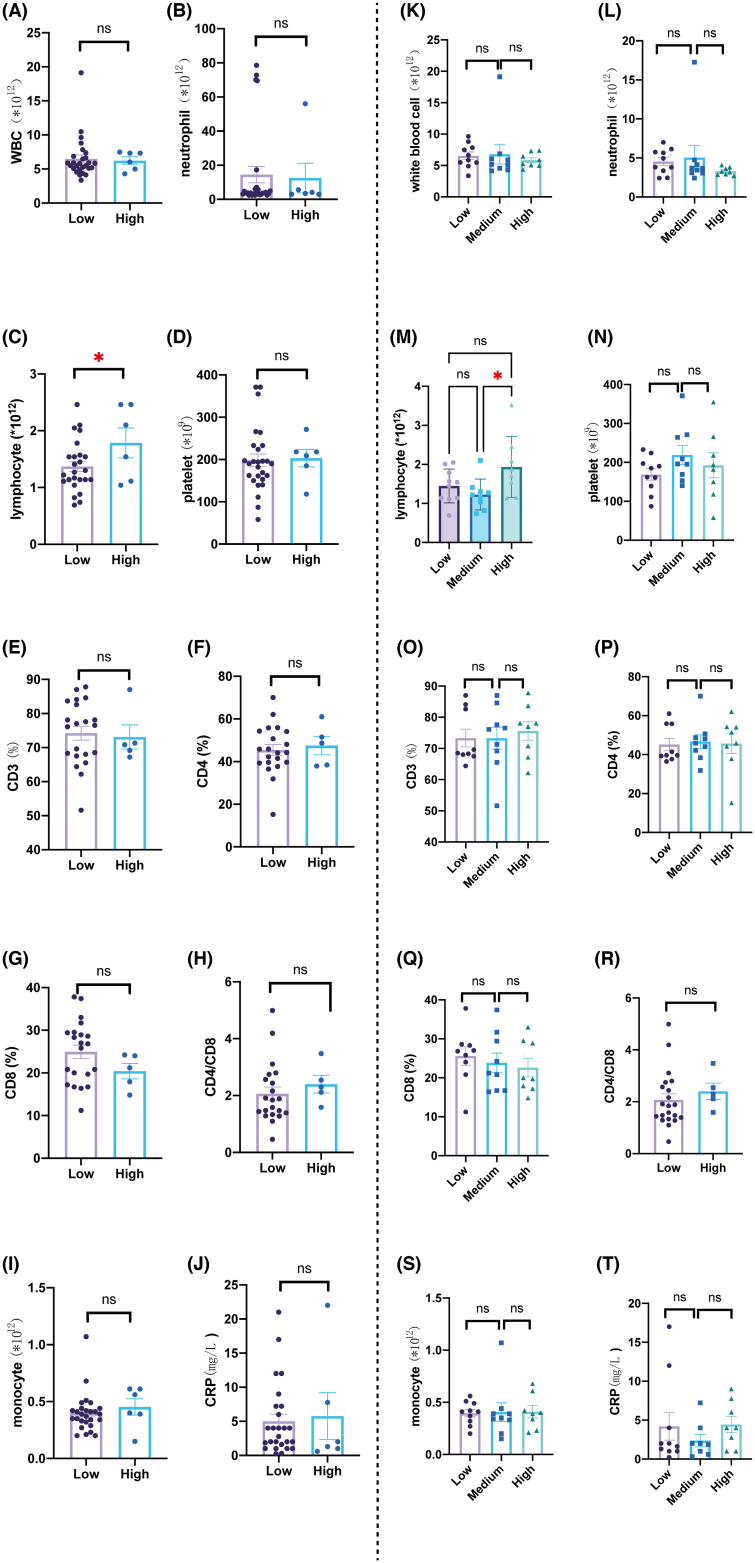
Blood cell count and C‐reactive protein differences among patients with different epinephrine levels. *p < 0.05. (A–J) Blood cell count and C‐reactive protein differences between two‐grouped patients according to epinephrine levels. (K–T) Blood cell count and C‐reactive protein differences among three‐grouped patients according to epinephrine levels.

As epinephrine mainly acts as an endocrine hormone, it is likely to cause blood cell count disturbance. So we also plotted its relationship with the frequently tested blood cell count as well as C‐reactive protein (an index for inflammation). Analyses showed that those with the highest epinephrine presented with the highest lymphocyte count (Table S1, Figure 3C,M).

**FIGURE 4:**
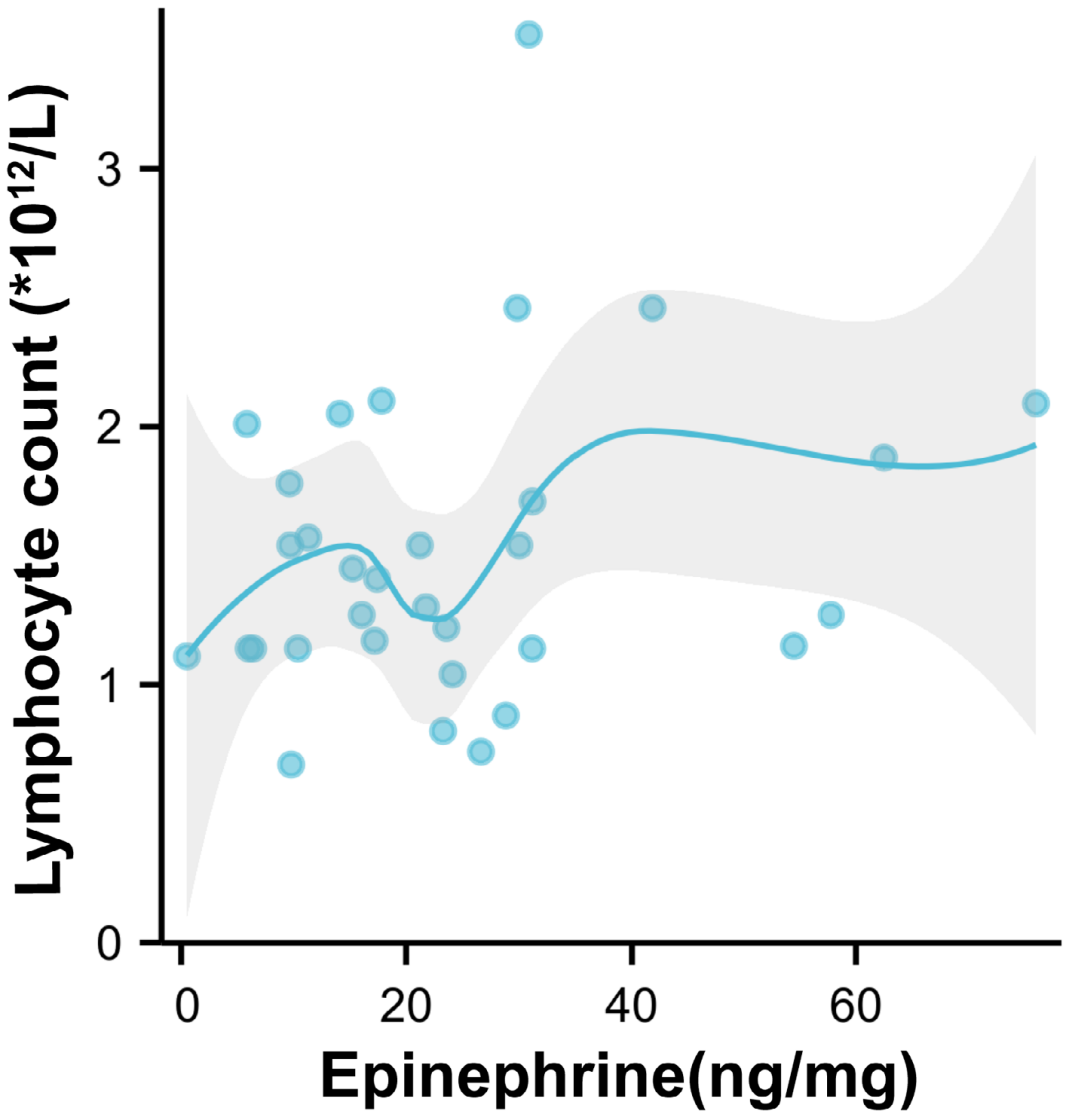
The scatter diagram digging into the relationship of tissue epinephrine (ng/mg) and lymphocyte count (×10^12^/L).

Those with medium epinephrine showed the lowest lymphocyte level (1.23 ± 0.39 × 10^12^/L), significantly different from that (1.94 ± 0.78 × 10^12^/L) in the highest epinephrine group (*p* < 0.05), which was also revealed in the scatter diagram with curve‐fitting (Figure 4), in accordance with Figure 3M.

### 3.4 Circulating lymphocytes compensated for the correlation of epinephrine and PDAC survival

We just confirmed the association between epinephrine with PDAC survival and circulating lymphocyte count. But whether lymphocyte count contributes to the correlation between epinephrine and PDAC is still unknown.

We used unicox regression and multicox regression to pinpoint the role of lymphocytes. Lymphocytes were equally divided into three group levels (<1.16 × 10^12^/L,1.16–1.6 × 10^12^/L, >1.6 × 10^12^/L) in the Cox regression. Lymphocyte group presented as a protective factor for the survival of PDAC patients as HRs were 0.40 (0.10–1.62) and 0.55 (0.1364–2.19) for the high and medium lymphocyte group compared with low lymphocyte group, even though no statistical significance was shown (Table S1, Figure S1). Median survival time was 424 days in patients with low lymphocyte level, while it was not reached in the high and medium lymphocyte groups.

When we divided epinephrine using dichotomy method, HR of epinephrine for death was 11.03 (2.51–48.57), and lymphocyte adjustment increased the HR by 99.1% (Table 2). When we divided epinephrine using trichotomy method, HR for high epinephrine versus medium epinephrine was 7.66 (0.92–63.98) and lymphocyte adjustment increased the HR by 15.8% (Table 3). Likelihood ratio test value and C‐index of the model were also all increased after being adjusted by lymphocyte. These data indicate that lymphocyte was an intermediary factor between epinephrine and PDAC death, as lymphocyte reduced the effect of epinephrine on mortality, conducted from the increase of HR after being adjusted by lymphocyte group.

**TABLE 2:** Model comparisons for the basic model (two‐grouped epinephrine) with and without lymphocyte.

		Uni‐cox	Adjusted by lymphocyte group^a^
HR (95% CI)	High versus Low	11.03 (2.51–48.57)**	21.96 (3.92–123.11)***
Likelihood ratio comparisons		10.08	14.9
C‐index		0.68 (SE = 0.062)	0.8 (SE = 0.05)

Abbreviation: SE, standard error.

^a^ Lymphocytes were divided into three group levels (*n* = 11 for lymphocyte <1.16*10^12^/L, *n* = 11 for lymphocyte 1.16–1.6 × 10^12^/L, *n* = 10 for lymphocyte >1.6 × 10^12^/L) in the cox regression.

** *p* < 0.01;

*** *p* < 0.001.

**TABLE 3:** Model comparisons for the basic model (three‐grouped epinephrine) with and without lymphocyte.

		Uni‐cox	Adjusted by lymphocyte group^a^
HR (95% CI)	High versus medium	7.66 (0.92–63.98)	8.87 (1.00–78.47)*
	Low versus medium	4.71 (0.55–40.37)	4.30 (0.49–38.00)
Likelihood ratio comparisons		5.34	7.34
C‐index		0.69 (SE = 0.07)	0.73 (SE = 0.07)

Abbreviation: SE, standard error.

^a^ Lymphocytes were divided into three group levels (*n* = 11 for lymphocyte <1.16 × 10^12^/L, *n* = 11 for lymphocyte 1.16–1.6 × 10^12^/L, *n* = 10 for lymphocyte >1.6 × 10^12^/L) in the cox regression.

* *p* < 0.05.

The high HR of high versus medium and low versus medium reconfirmed the inverted U‐shaped relationship between epinephrine and PDAC survival, in accordance with Figures 1B and 2.

## 4 DISCUSSION

This this the first study that analyzed the correlation of epinephrine with PDAC survival using epinephrine as 3 grouped levels in human tissue. An inverted U‐shaped relationship was demonstrated between epinephrine and PDAC death. The outcome is consistent with Liu's et al. in vivo study of epinephrine administration on cancer.^5^ Low dose of epinephrine (2 mg/kg) can inhibit tumor progression while high dose of epinephrine (6 mg/kg) promotes tumor progression. And as epinephrine is also responsible for stress, this also indicates low levels of stress can be body protective while too much stress may damage the body function.

But different from Liu's et al. study, we haven't found differences in monocyte (precursor of macrophage). And even though patients with high epinephrine accompanies by high lymphocyte count, it seems like lymphocyte increase was a compensation pathway to reduce the correlation between epinephrine and mortality as HRs increased after being adjusted by lymphocyte level (Tables 2 and 3).

Even though lymphocyte was not shown as a factor for survival prediction in our study (Figure S1), the lymphocyte population fluctuation caused by epinephrine may be a specific lymphocyte subset. Tests on more detailed lymphocyte subsets and functions should be done for further study.

There are some limitations of this paper. First, the patient population was not big enough. Second, we had not collected the epinephrine of blood samples or lymphocyte subset distribution of PDAC tissues. We would do further research using blood epinephrine/lymphocyte and tissue epinephrine/lymphocyte comprehensively of a larger sample in the future.

## 5 CONCLUSIONS

All in all, our study found that epinephrine played an anti‐tumor or pro‐tumor effect depending on the specific concentration. Circulating lymphocyte count was elevated and might acted as a compensation pathway to reduce the pro‐tumor effect of epinephrine to PDAC.

## AUTHOR CONTRIBUTIONS

**Bing‐Xue Li:** Conceptualization (lead); data curation (equal); formal analysis (lead); investigation (lead); methodology (equal); writing – original draft (lead). **Xiao‐Cen Zhu:** Data curation (equal); formal analysis (supporting); methodology (equal); writing – review and editing (equal). **Xia‐Xing Deng:** Funding acquisition (lead); project administration (lead); supervision (lead); writing – review and editing (lead).

## CONFLICT OF INTEREST STATEMENT

There are no competing interests to declare.

## ETHICS STATEMENT

This study was approved by the Institutional Review Board of Ruijin Hospital, Shanghai Jiaotong University School of Medicine.

## Supporting information


Table S1.
Table S2.
Figure S1.

## Data Availability

All data that supports the findings of the study are included in this manuscript.
