# Supplementary material for: The inverted U‐shaped relationship between epinephrine and pancreatic ductal adenocarcinoma patients' survival with compensation of lymphocyte
Source: Cancer Med. 2024 Apr 4;13(7):e7164. doi: 10.1002/cam4.7164 (PMC10993700; doi:10.1002/cam4.7164)
Supplement: Supplementary file 1 — Table S1. Table S2. Figure S1. [file CAM4-13-e7164-s001.docx]

**Table S1 Basic characteristics of patients grouped by different levels of epinephrine**

|  | **Low epinephrine**  **n=11** | **Medium epinephrine**  **n=10** | **High epinephrine**  **n=11** | ***P*** |
| --- | --- | --- | --- | --- |
| **Age, y** | 67.00[62.00,72.25] | 64.50[52.25,68.00] | 63.00[59.00,66.00] | 0.283 |
| **Gender** |  |  |  | 0.788 |
| Male | 6 (60.0) | 5 (50.0) | 4 (44.4) |  |
| Female | 4 (40.0) | 5 (50.0) | 5 (55.6) |  |
| **BMI, kg/m^2^** | 23.19±2.25 | 22.89±3.18 | 22.69±3.29 | 0.93 |
| **Pathology** |  |  |  | 0.133 |
| Poor | 3 (30.0) | 0 (0.0) | 1 (10.0) |  |
| Well/moderate | 7 (70.0) | 10 (100.0) | 9 (90.0) |  |
| **TNM** |  |  |  | 0.409 |
| I | 1 (9.1) | 1 (10.0) | 2 (18.2) |  |
| II | 4 (36.4) | 4 (40.0) | 7 (63.6) |  |
| III | 4 (36.4) | 4 (40.0) | 1 (9.1) |  |
| IV | 2 (18.2) | 1 (10.0) | 1 (9.1) |  |
| **Perineural invasion** |  |  |  | NA |
| Yes | 11 (100.0) | 10 (100.0) | 11 (100.0) |  |
| No | 0(0) | 0(0) | 0(0) |  |
| **Vascular invasion** |  |  |  | 0.505 |
| Yes | 9 (81.8) | 6 (60.0) | 7 (63.6) |  |
| No | 2 (18.2) | 4 (40.0) | 4 (36.4) |  |
| **Type of surgery** |  |  |  | 0.144 |
| PD | 5 (45.45) | 6 (60.0) | 2 (18.2) |  |
| DP | 5 (45.45) | 2 (20.0) | 8 (72.7) |  |
| TP | 1 (9.1) | 2 (20.0) | 0 (0.0) |  |
| MP | 0 (0.0) | 0 (0.0) | 1（9.1） |  |
| **R** |  |  |  | 0.373 |
| R0 | 10 (90.9) | 10 (100.0) | 11 (100.0) |  |
| R1 | 1 (9.1) | 0 (0.0) | 0 (0.0) |  |
| **CA19-9, U/ml** | 161.85[57.40,1281.28] | 444.15[209.93,738.55] | 94.45[15.95,835.78] | 0.403 |
| **While blood cell, 10^9^/L** | 5.85[5.47,7.66] | 5.54[4.70,6.66] | 5.89[5.18,6.89] | 0.795 |
| **Neutrophil , 10^9^/L** | 4.95[3.58,5.95] | 3.69[3.19,4.59] | 3.64[3.06,30.05] | 0.807 |
| **Platelet, 10^9^/L** | 174.73±48.41 | 213.00±70.73 | 195.00±76.56 | 0.426 |
| **Lymph, 10^9^/L** | 1.45±0.43 | 1.23±0.39 | 1.94±0.78 | **0.071** |
| **CD3^+^, %** | 68.50[68.00,82.60] | 76.10[69.20,77.60] | 77.55[69.90,79.50] | 0.783 |
| **CD3^+^CD4^+^, %** | 39.70[39.30,55.80] | 45.90[42.20,48.70] | 48.65[42.63,54.15] | 0.791 |
| **CD3^+^CD8^+^, %** | 25.59±7.08 | 23.83±7.50 | 22.62±6.79 | 0.694 |
| **CD3^+^CD4^+^/CD3^+^CD8^+^** | 1.48[1.33,2.16] | 1.91[1.44,2.44] | 2.34[1.78,2.84] | 0.472 |
| **Mono, 10^12^/L** | 0.41[0.34,0.45] | 0.37[0.33,0.43] | 0.40[0.32,0.52] | 0.737 |
| **CRP(mg/L)** | 2.00[1.15,3.00] | 2.00[1.00,4.00] | 6.00[3.39,8.39] | 0.111 |
| Abbreviation: y, year; BMI, body mass index; PD, pancreatoduodenectomy; DP, distal pancreatectomy; TP, total pancreatectomy; MP, middle segment pancreatectomy. | | | |  |

**Table S2 Unicox regression of lymphocyte group for PDAC survival**

|  |  | Uni-cox |
| --- | --- | --- |
| HR（95% CI) | High vs Low | 0.40 (0.10-1.62) |
|  | Medium vs Low | 0.55 (0.1364-2.19) |
| Likelihood ratio comparisons | | 1.85 |
| C-index | | 0.615 (se = 0.07 ) |
| Lymphocytes were divided into three group levels [n=11 for lymphocyte <1.16*10^12^/L, n=11 for lymphocyte 1.16-1.6*10^12^/L, n=10 for lymphocyte >1.6*10^12^/L] in the cox regression.  se: standard error | | |


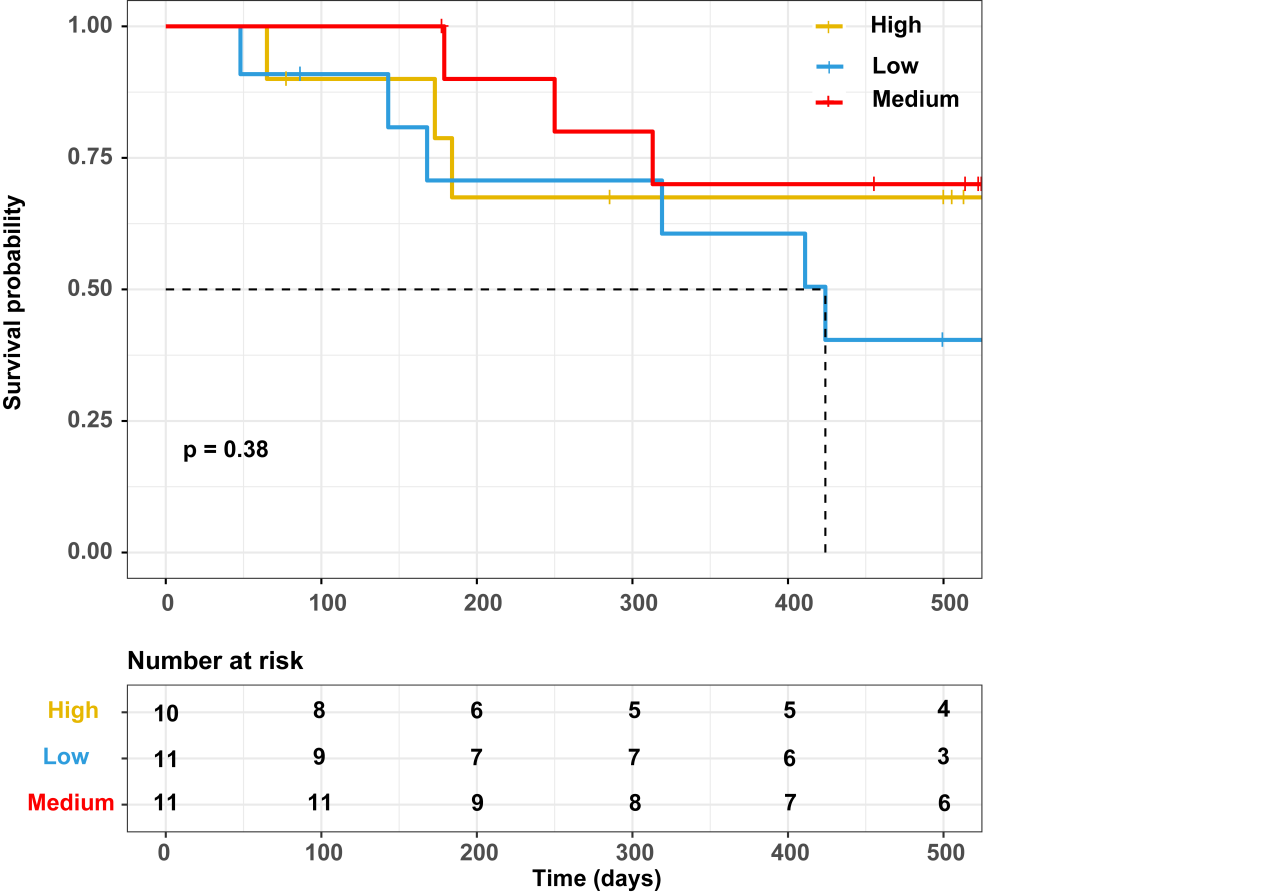


**Figure S1 Relationship of lymphocyte group with mortality cumulative risk.** Lymphocytes were divided into three group levels: n=11 for lymphocyte <1.16*10^12^/L, n=11 for lymphocyte 1.16-1.6*10^12^/L, n=10 for lymphocyte >1.6*10^12^/L.
